# Maternal postpartum depressive symptoms partially mediate the association between preterm birth and mental and behavioral disorders in children

**DOI:** 10.1038/s41598-022-04990-w

**Published:** 2022-01-18

**Authors:** Polina Girchenko, Rachel Robinson, Ville Juhani Rantalainen, Marius Lahti-Pulkkinen, Kati Heinonen-Tuomaala, Sakari Lemola, Dieter Wolke, Daniel Schnitzlein, Esa Hämäläinen, Hannele Laivuori, Pia M. Villa, Eero Kajantie, Katri Räikkönen

**Affiliations:** 1 Institute of Psychology and Logopedics, Faculty of Medicine, University of Helsinki, (Haartmaninkatu 3), P.O BOX 21, 00014 Helsinki, Finland; 2 National Institute for Health and Welfare, Helsinki, Finland; 3 Queen’s Medical Research Institute, University of Edinburgh, Edinburgh, UK; 4 Psychology/Welfare Sciences, Faculty of Social Sciences, Tampere University, Tampere, Finland; 5 Department of Psychology, University of Warwick, Coventry, UK; 6 Department of Psychology, Bielefeld University, Bielefeld, Germany; 7 Leibniz University of Hannover, Hannover, Germany; 8 IZA Bonn, Bonn, Germany; 9 Department of Clinical Chemistry, University of Eastern Finland, Kuopio, Finland; 10 Department of Obstetrics and Gynecology, Tampere University Hospital, Tampere, Finland; 11 Center for Child, Adolescent and Maternal Health Research, Faculty of Medicine and Health Technology, Tampere University, Tampere, Finland; 12 Medical and Clinical Genetics, University of Helsinki and Helsinki University Hospital, Helsinki, Finland; 13 Institute for Molecular Medicine Finland, Helsinki Institute of Life Science, University of Helsinki, Helsinki, Finland; 14 Obstetrics and Gynaecology, Helsinki University Hospital and University of Helsinki, Helsinki, Finland; 15 National Institute for Health and Welfare, Public Health Promotion Unit, Helsinki, Finland; 16 University of Oulu, Oulu, Finland

**Keywords:** Psychology, Risk factors

## Abstract

Preterm birth has been linked with postpartum depressive (PPD) disorders and high symptom levels, but evidence remains conflicting and limited in quality. It remains unclear whether PPD symptoms of mothers with preterm babies were already elevated before childbirth, and whether PPD symptoms mediate/aggravate the effect of preterm birth on child mental disorders. We examined whether preterm birth associated with maternal PPD symptoms, depressive symptoms trajectories from antenatal to postpartum stage, and whether PPD symptoms mediated/aggravated associations between preterm birth and child mental disorders. Mothers of preterm (n = 125) and term-born (n = 3033) children of the Prediction and Prevention of Preeclampsia and Intrauterine Growth Restriction study reported depressive symptoms four times within 8 weeks before and twice within 12 months after childbirth. Child mental and behavioral disorder diagnoses until age 8.4–12.8 years came from medical register. Preterm birth associated with higher PPD symptoms (mean difference = 0.19 SD, 95% CI 0.01, 0.37, *p* = 0.04), and higher odds (odds ratio = 2.23, 95% CI 1.22, 4.09, *p* = 0.009) of the mother to belong to a group that had consistently high depressive symptoms levels trajectory from antenatal to postpartum stage. PPD symptoms partially mediated and aggravated the association between preterm birth and child mental disorders. Preterm birth, maternal PPD symptoms and child mental disorders are associated, calling for timely prevention interventions.

## Introduction

Preterm birth (< 37 completed weeks of gestation) is the leading cause of perinatal morbidity and mortality^1^. It poses a risk for neurosensory impairments, and for physical, mental and behavioural disorders later in life^2–5^. Preterm birth also induces substantial challenges and distress among the parents of the preterm infants^6^. Hence, preterm birth has been implicated as a risk factor for maternal postpartum depression (PPD)^7^.

However, high-quality studies on the association between preterm birth and maternal PPD have been scarce. Using the Newcastle–Ottawa Scale (NOS)^8^, we systematically assessed the quality of evidence of previous studies testing the association between preterm birth and maternal PPD (Tables ST1 and ST2). The NOS assessment revealed the limited quality of the available evidence: of the 26 reviewed cohort and cross-sectional studies, 16 (61.5%) were found of low quality, five (19.2%) of moderate and five (19.2%) of high quality (ST1, ST2). Of the five high-quality studies, three were population-based register studies with focus on maternal PPD disorder diagnoses up to 12 months after childbirth^9–11^. Two of the register studies^10,11^ found an association between preterm birth and maternal PPD, and one^9^ reported a null association. The other two high-quality studies were cohort studies with focus on maternal PPD symptoms. One of the cohort studies did not find an association between preterm birth and maternal PPD symptoms two months after childbirth^12^, while the other did, but used one non-validated question addressing maternal low mood, depression and hopelessness 2–4 months after childbirth as an indicator of PPD^13^. This calls for further high-quality studies on preterm birth and PPD symptoms measured using validated scales. Furthermore, as depressive symptoms show high continuity from the antenatal to the postpartum stage^14^, further studies are also needed that examine if PPD symptoms of mothers with preterm babies were already elevated in the antenatal stage.

Apart from the implicated association with preterm birth, maternal PPD has been also linked with the child’s higher risk for mental and behavioral disorders^14,15^, which suggests that PPD may be on a pathway between preterm birth and mental and behavioral disorders in children. One previous study has reported that maternal major depressive disorder (MDD) diagnosis between childbirth and child’s assessment in early childhood mediated the association between lower gestational age and child’s risk for anxiety disorders^16^. Another study reported that maternal PPD symptoms 9 months postpartum did not mediate the association between preterm birth and child’s cognitive function in early childhood^17^. However, we are not aware of studies that have tested whether maternal PPD symptoms mediate the associations between preterm birth and child mental and behavioral disorders. It also remains unknown if maternal PPD aggravates the association of preterm birth with these child mental health outcomes.

We examined in a large pregnancy cohort of Finnish women and their children if preterm birth was associated with maternal PPD symptoms and probable clinical PPD (symptoms scores above a threshold that have been shown to be sensitive and specific in identification of individuals meeting the diagnostic MDD criteria^18^) from the childbirth until 12 months postpartum. Further, to unravel if the depressive symptoms levels of women with a preterm baby were high already in the antenatal stage, we studied if preterm birth was associated with depressive symptoms trajectories from the antenatal to the postpartum stage. We also examined if these associations were independent of maternal mood disorders diagnosed before childbirth, and examined if maternal PPD symptoms mediated the association between preterm birth and mental and behavioral disorders in the children followed-up from birth until mid-childhood. Finally, we examined if the effects of preterm birth and maternal PPD on mental and behavioral disorders in the children were additive. We took into account important covariates and assessed whether maternal obstetric complications associated with the risk of preterm delivery, namely preeclampsia, placenta previa, chorionamnionitis, premature rupture of membranes and mode of delivery^19^, modified the associations of preterm birth with maternal PPD, depressive symptoms trajectories and child mental and behavioral disorders.

## Results

Descriptive characteristics of the preterm and term born children and their mothers are shown in Table 1. Of the preterm born children 88% (n = 110) were born moderately or late preterm (32^+0^–36^+6^ gestational weeks) and 12% (n = 15) were born very or extremely preterm (< 32^+0^ gestational weeks). Preterm born compared with term born children were more often boys. Mothers of preterm born compared with term born children more often had a pregnancy complicated by preeclampsia and premature rupture of membranes, they more often delivered via Caesarean section, especially urgent/emergency Caesarean section, and reported higher levels of antenatal depressive symptoms 8 weeks before childbirth (Table 1). Associations between the covariates and maternal PPD symptoms and mental and behavioral disorders in children are presented in Table ST4.

**Table 1:** Characteristics of the sample.

	Mean (SD) or N (%)		*P*
	Term birth (N = 3033)	Preterm birth (N = 125)	
**Child characteristics**			
Child sex			
Boy	1552 (51.2%)	77 (61.6%)	0.02
Girl	1481 (48.8%)	48 (38.4%)	
Data not available	0	0	
Gestational age at birth, weeks	40.0 (1.1)	35.0 (2.1)	< 0.0001
Data not available	0	0	
Follow-up length years (Median, IRQ)	9.7 (9.5–10.7)	9.7 (9.6–10.9)	0.65
Data not available	0	0	
Mental and behavioral disorder diagnosis			0.02
No	2720 (89.7%)	104 (82.2%)	
Yes	313 (10.3%)	21 (16.8%)	
Data not available	0	0	
**Maternal characteristics**			
Maternal age at delivery, years	31.8 (4.7)	31.7 (4.8)	0.87
Data not available	0	0	
Education level			0.81
Upper secondary or less	1226 (40.5%)	52 (41.6%)	
Tertiary	1801 (59.5%)	73 (58.4%)	
Data not available	6 (0.2%)	0	
Smoking or alcohol use at any point during pregnancy			0.81
No	2347(77.4%)	99 (79.2%)	
Yes	648 (22.6%)	24 (20.8%)	
Data not available	38 (1.3%)	2 (1.6%)	
Preeclampsia (O11, O14, O15)			< 0.0001
No	2939 (96.8%)	94 (77.7%)	
Yes	98 (3.2%)	27 (22.3%)	
Data not available	0	0	
Chorioamniotitis (O41.1)			0.10
No	3018 (99.5%)	123 (98.4%)	
Yes	15 (0.5%)	2 (1.6%)	
Data not available	0	0	
Placenta previa (O44)			0.10
No	3016 (99.8%)	121 (99.2%)	
Yes	5 (0.2%)	1 (0.8%)	
Data not available	12 (0.4%)	3 (2.4%)	
Premature rupture of membranes (O42)			< 0.0001
No	2941 (97.0%)	92 (73.6%)	
Yes	92 (3.0%)	33 (26.4%)	
Data not available	0	0	
Mode of delivery			< 0.0001
Vaginal	2532(83.8%)	76 (61.3%)	
Caesarean section	490 (16.2%)	48 (38.7%)	
Elective Caesarean section	192 (6.4%)	13 (10.5%)	
Urgent or emergency Caesarean section	298 (9.9%)	35 (28.2%)	
Data not available	11 (0.4%)	1 (0.8%)	
Antenatal depressive symptoms 6–8 weeks before delivery (continuous CES-D score)	11.8 (7.2)	13.3 (8.4)	0.02
Data not available	130 (4.3%)	5 (4.0%)	
Antenatal probable clinical depression 6–8 weeks before delivery (CES-D ≥ 16)			0.04
No	2182 (75.2%)	80 (66.7%)	
Yes	721 (24.8%)	40 (32.3%)	
Data not available	1407 (32.2%)	65 (34.4%)	
Postpartum depressive symptoms up to 12 months after childbirth (continuous CES-D score)	10.2 (7.0)	11.7 (7.2)	0.02
Data not available	0	0	
Postpartum probable clinical depression up to 12 months after childbirth (CES-D ≥ 16)			0.34
No	2458 (81.0%)	97 (77.6%)	
Yes	575 (19.0%)	28 (22.4%)	
Data not available	0	0	
Mood disorder diagnoses before childbirth			0.88
No	2896 (95.5%)	119 (95.2%)	
Yes	137 (4.5%)	6 (4.8%)	
Data not available	0	0	

CES-D refers to Center for Epidemiologic Studies Depression Scale; O-codes refer to International Statistical Classification of Diseases and Related Health-problems 10th revision.

### Preterm birth and maternal PPD symptoms

In unadjusted and adjusted models, preterm birth was associated with higher levels of maternal PPD symptoms. When we excluded women with mood disorders diagnosed before childbirth from the analyses, the associations remained significant (Table 2). Preterm birth was not associated with higher odds of maternal probable clinical PPD (Table 2).

**Table 2:** Associations between preterm versus term birth and maternal postpartum depressive symptoms (PPD).

Preterm versus term birth	Level of PPD symptoms			Probable clinical PPD			Latent classes of women based on antenatal and PPD symptoms					
							Consistently moderate versus low			Consistently high versus low		
	Mean difference (SD units)	95% Confidence Interval	*P*	Odds Ratio	95% Confidence Interval	*P*	Odds Ratio	95% Confidence Interval	*P*	Odds Ratio	95% Confidence Interval	*P*
Model 1	0.22	0.04, 0.40	0.02	1.23	0.80, 1.90	0.34	1.70	0.96, 2.99	0.07	2.25	1.24, 4.10	0.008
Model 2	0.19	0.01, 0.37	0.04	1.14	0.74, 1.77	0.55	1.69	0.95, 3.00	0.07	2.23	1.22, 4.09	0.009
Model 3	0.19	0.01, 0.38	0.04	1.16	0.74, 1.83	0.52	1.65	0.93, 2.93	0.09	2.25	1.22, 4.15	0.009

Model 1 is unadjusted; Model 2 is adjusted for maternal age at delivery, maternal education, maternal smoking and/or alcohol use at any time during pregnancy, delivery mode and child sex; Model 3 is a sensitivity analyses: Model 2 where women with mood disorder diagnoses before childbirth are excluded.

### Preterm birth and maternal depressive symptoms trajectories between antenatal and postpartum stage

The optimal LCA solution identified three groups of women who differed in depressive symptoms levels trajectories between the antenatal and postpartum stage (Table ST5). In all three groups, levels of depressive symptoms showed high stability: the levels were consistently low (n = 633, 20.0%), moderate (n = 1696, 53.7%) or high (n = 829, 26.3%) between the antenatal and postpartum stage (Fig. S1). Women in the group with consistently high levels of depressive symptoms between the antenatal and postpartum stage had depressive symptoms scores that were consistently at or above the probable clinical cutoff (CES-D ≥ 16) (Fig. S1).

In unadjusted and adjusted models, preterm birth was associated with significantly higher odds of the mother to belong to the group who had a consistently high versus low symptoms levels trajectory from the antenatal to postpartum stage (Table 2). The corresponding odds of the mother to belong to the group who had a consistently moderate versus low symptoms levels trajectory from the antenatal to postpartum stage was not statistically significant (Table 2).

### Maternal PPD symptoms as a mediator between preterm birth and mental and behavioral disorders in children

**Figure 1:**
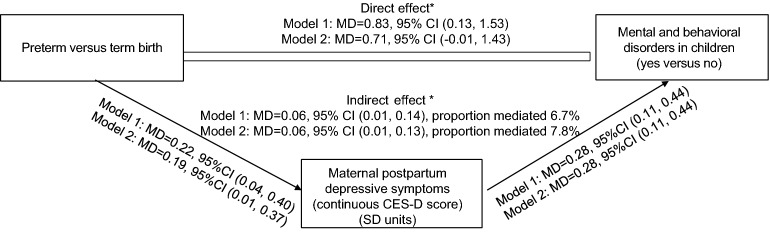
Mediation analysis showing that the effect of preterm birth on mental and behavioral disorders in children until age 8.4–12.8 years, is partially mediated via maternal postpartum depressive symptoms. Model 1 refers to unadjusted associations and Model 2 to associations adjusted for maternal age at delivery, education, smoking and/or alcohol use at any time during pregnancy, delivery mode and child sex and age at diagnosis. The effects are shown as mean differences (MD) and their 95% Confidence Intervals (95% CI).

Table 3 shows that both preterm birth and higher levels of maternal PPD symptoms were associated with significantly higher hazards of mental and behavioral disorders in children. Mediation analyses indicated that maternal PPD symptoms partially mediated the association between preterm birth and mental and behavioral disorders in children (Fig. 1) with the effect size proportion mediated being 6.7% in unadjusted and 7.8% in adjusted models.

**Table 3:** Associations between preterm birth and maternal postpartum depressive symptoms (PPD) with mental and behavioral disorders in children in a follow-up to 8.4–12.8 years of age.

	Mental and behavioral disorders in children		
	Hazard Ratio	95% Confidence Interval	P
**Preterm versus term birth**			
Model 1	1.61	1.04, 2.51	0.03
Model 2	1.57	1.00, 2.45	0.05
**Maternal PPD symptoms (continuous CES-D score) (SD units)**			
Model 1	1.26	1.14, 1.41	< 0.0001
Model 2	1.24	1.12, 1.38	< 0.0001
**Maternal probable clinical PPD (CES-D ≥ 16 versus CES-D < 16)**			
Model 1	1.51	1.18, 1.93	0.0009
Model 2	1.47	1.15, 1.88	0.002

Model 1 is unadjusted; Model 2 is adjusted for maternal age at delivery, education, smoking and/or alcohol use at any time during pregnancy, delivery mode and child sex. CES-D refers for Center for Epidemiologic Depression Scale.

### Additive effects of preterm birth and maternal PPD symptoms on mental and behavioral disorders in children

**Figure 2:**
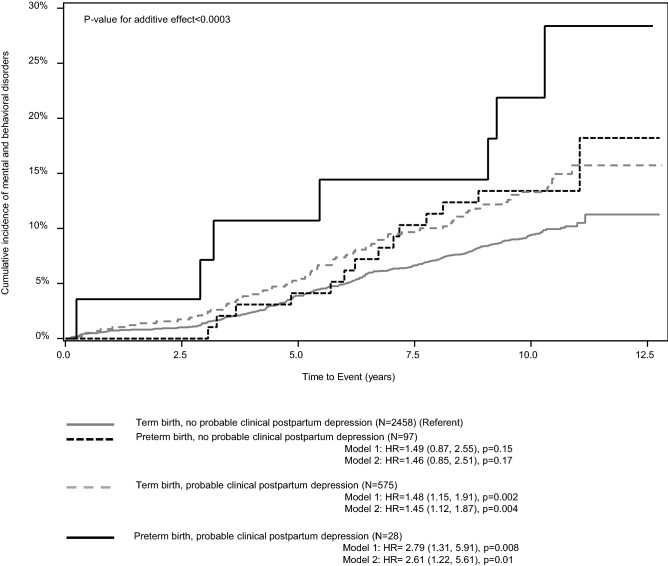
Additive effects of preterm birth and maternal postpartum depressive symptoms on mental and behavioral disorders in children. The lines represent cumulative incidence rates of mental and behavioral disorders until age 8.4–12.8 years in children born at term and whose mother reported no probable clinical postpartum depression (referent), (**a**) in children born preterm and whose mothers reported no probable clinical postpartum depression, (**b**) in children born at term and whose mother reported probable clinical postpartum depression, and (**c**) in children born preterm and whose mother reported probable clinical postpartum depression. HR refers to hazard ratio and 95% CI to 95% Confidence Interval. Model 1 refers to unadjusted associations and Model 2 to associations adjusted for maternal age at delivery, education, smoking and/or alcohol use at any time during pregnancy, delivery mode and child sex. *P* value for linearity refers to the additive effect of preterm birth and maternal postpartum depression (0 = term, no postpartum depression, 1 = preterm, no postpartum depression or term, postpartum depression, 2 = preterm, postpartum depression).

Figure 2 shows that compared to term-born children born whose mothers had no probable clinical PPD, the hazard of mental and behavioral disorders was 46% higher for children born preterm and whose mother reported no probable clinical PPD, 45% higher for children born at term and whose mothers reported probable clinical PPD, and 161% higher for children born preterm and whose mothers reported probable clinical PPD. The effects of preterm birth and maternal PPD were additive, as the child’s hazard for mental and behavioral disorders increased linearly according to the number of these exposures (0 = term, no PPD, 1 = term and PPD or preterm and no PPD, 2 = preterm and PPD) (Fig. 2).

### Effect modification by maternal obstetric complications and mode of delivery

None of the associations presented above were modified by maternal preeclampsia, premature rupture of membranes or delivery via elective or urgent/emergency Caesarian section vs. vaginal delivery (Table ST6). Table 1 shows that there were too few cases to study effect modification by placenta previa and chorioamnionitis.

## Discussion

This study showed that mothers whose babies were born preterm had higher levels of PPD symptoms up to 12 months postpartum. This association remained significant even after we excluded mothers with mood disorder diagnosed before the childbirth. Preterm birth was not associated with maternal probable clinical PPD, pointing to only subtle differences in the levels of depressive symptoms between the mothers who gave birth to preterm and term babies. However, when we took into account the continuity of maternal depressive symptoms from the antenatal to the postpartum periods, mothers who gave birth to preterm babies had over twofold higher odds to belong to the group displaying consistently high depressive symptoms, which were at or above the probable clinical cutoff from the antenatal to the postpartum stage. These findings suggest that even though the differences appear to be subtle in the levels of PPD symptoms between mothers who gave birth to preterm and term babies, these differences are aggravated by high levels of antenatal depressive symptomatology persisting to the postpartum stage.

These findings are consistent with the one high-quality cohort study from the US, which found that preterm birth was associated with maternal feelings of low mood, depression and hopelessness 2–4 months after childbirth^13^. They are also in partial agreement with the other high-quality cohort study from Greece, which found no associations between preterm birth and maternal probable clinical PPD measured two months after childbirth with the EPDS^12^. However, both of these studies used a different measure to capture PPD symptoms than we did. Neither of these studies followed the women longer than 2–4 months after childbirth, nor studied trajectories of depressive symptoms from the antenatal to the postpartum stage, as we did, therefore, our study adds to high-quality evidence.

Our findings indicate that maternal PPD symptoms partially mediate the association between preterm birth and the risk of mental and behavioral disorders in children. The effect size proportion mediated is, however, less than 8%, suggesting that maternal PPD symptomatology is only a modest mediator of this association. At the same time, both preterm birth, maternal PPD symptoms and probable clinical PPD are associated with the child’s higher hazard of mental and behavioral disorders, and their effects appear to be additive. While we and others have also shown that preterm birth^2–5^ and maternal PPD^14,20^ were associated with higher mental health risks in children, we are not aware of previous reports showing that these risks were additive.

Our results suggest that while it is difficult to prevent preterm birth in itself^21^, it would be feasible to target maternal PPD symptoms. The continuity of depressive symptoms from the antenatal to the postpartum stage, however, highlights that preventive interventions that focus on maternal depressive symptomatology should start already in pregnancy. Further benefits may be gained by postpartum interventions, as it has been shown that intervention programs that focus on decreasing stress in the parents of preterm infants also reduce the risk of maternal PPD^22,23^. Addressing PPD in women who gave birth to preterm babies should alleviate the adverse effects of preterm birth: even a small reduction of the adverse effect of preterm birth should result in considerable public health benefits on a population level. Furthermore, reduction of maternal PPD symptoms in women who gave birth to preterm babies should lessen additive effects of preterm birth and PPD on children’ mental health risks.

The ability to verify that the associations between preterm birth and maternal PPD symptoms were not explained by maternal history of mood disorders and the ability to take into account the continuity of depressive symptomatology from the antenatal to the postpartum stage, both strong predictors of maternal PPD^10,14,24^, are key strengths in this study. We were also able to show that the associations between preterm birth and maternal PPD symptoms, depressive symptoms trajectories and child outcomes were not modified by maternal preeclampsia, premature rupture of membranes or mode of delivery. Other study strengths include prospective study design, large and well-characterized sample, longitudinal measurement of depressive symptoms from the antenatal to the postpartum stage, which allowed us to identify maternal antenatal depressive symptoms before preterm and term delivery, data on maternal mood and child mental and behavioral disorder diagnoses extracted from nationwide registers and null data attrition in child follow-up.

However, our study does not unravel the mechanisms underlying the association between preterm birth and maternal PPD symptoms, and mechanisms underlying their association with increased risk of mental and behavioral disorders in children. While data attrition in the child follow-up was null, our study suffers from selective dropout, as the women who participated in the postpartum follow-up and reported PPD symptoms were older, used substances less, and less often had been diagnosed with mood disorders than the women who did not. However, even selective dropout has been found to only marginally alter predictive models^25^. Nevertheless, it may limit generalizability of our findings to less advantageous samples. A further limitation to generalizability is the high-resource Nordic setting. Finally, over 80% of the preterm births in our study were moderate or late preterm, which is in alignment with the population prevalence of moderate and late preterm births in Finland^26^. It is, hence, possible, that the associations might appear different in very and extremely preterm groups. It is also important to note that the first assessment of maternal PPD symptoms occurred at median 2.1 weeks after childbirth. However, in only 68 (2.2%) of the mothers in our sample the first PPD symptoms assessment occurred within the first 10 days after childbirth, a period known as the baby blues period^27^. Hence, since the vast majority of the mothers in our sample provided their first PPD assessment outside of baby blues period, and all second PPD assessments were way beyond this period, the baby blues period is unlikely to bias the associations. Young age of the children included in this study pertains to another limitation and precludes studying mental and behavioral disorders with peak age at onset beyond childhood, such as substance use disorders or schizophrenia-spectrum disorders^28^. Finally, our study is also limited by lacking data on paternal depressive symptoms, which may influence the associations. It has been previously demonstrated that in comparison to the fathers of term born children, fathers of preterm born children had elevated depressive symptoms 9 months after childbirth. These elevated paternal PPD symptoms, in turn, predicted child’s lower cognitive function in early childhood^17^. However, the symptoms did not mediate the associations between preterm birth and child’s cognitive function^17^.

In conclusion, our findings suggest that preterm birth and maternal PPD symptoms are associated. The continuity of depressive symptomatology from antenatal to postpartum period and its associations with preterm birth suggests that addressing depressive symptoms already in pregnancy may have beneficial effects on the risk of preterm birth, PPD and subsequently on child mental and behavioral disorders. Mediation and additive effects of preterm birth and maternal PPD on child’s higher risk for mental and behavioral disorders call for prevention interventions aimed at reducing PPD symptoms in women who gave preterm birth.

## Methods

### Participants

The Prediction and Prevention of Preeclampsia and Intrauterine Growth Restriction (PREDO) study is described in detail elsewhere^29^. In brief, it comprises 4777 mothers who gave birth to a singleton live-born child in Finland between 2006 and 2010. Recruitment took place at ten study hospitals in Southern/Eastern Finland at the first fetal ultrasound screening in early pregnancy. Three women have since withdrawn from the study. We excluded 6 mother–child dyads with unknown gestational age, 29 with missing Finnish Medical Birth Register (MBR) data, 178 with post-term birth (at gestational age > 42^+0^ weeks), and 4 whom we did not have data on child mental or behavioral disorders from the Finnish Care Register for Health Care (HILMO). Of the 4559 women, 3158 (69.3%) completed questionnaires on depressive symptoms twice up to 12 months postpartum, and of them 125 (4.0%) gave preterm birth.

Diagnoses of any mental disorder from birth until 31st December 2018, when the children were 8.4–12.8 (median 10.2, interquartile range (IRQ) 9.5–10.8) years of age, were available for all children of the 3158 women.

Characteristics of the women who did provide the data on PPD and those who did not are shown in the Table ST3. All participating women signed an informed consent. The consent enabled linkage to nationwide medical register data for the women and children using unique personal identification numbers assigned to all Finnish citizens and residents since 1971. The PREDO study protocol was approved by the Ethics Committees of the Helsinki and Uusimaa Hospital District and recruitment was conducted with permission from the participating study hospitals. All participants provided written informed consent. Consent of participating children was provided by parent(s)/legal guardian(s).

### Measures

#### Preterm birth.

Gestational age was derived from hospital records and/or MBR. We defined preterm birth as birth at 36^+6^ gestational weeks + days or less and term birth as birth between 37^+0^ and 41^+6^ gestational weeks + days.

#### Maternal PPD and antenatal depressive symptoms

The women completed the Center for Epidemiologic Studies Depression Scale (CES‐D)^18^ twice up to 12 months postpartum (time 1: median 2.1 weeks, IQR 2.0–2.4 weeks postpartum; time 2: 6.4 months, IQR 6.1–7.3 months postpartum). The 20 CES-D questions were rated on a scale from none (0) to all of the time (3). Higher scores indicate more depressive symptoms during the past week. We defined PPD symptoms as the mean of the two postpartum CES-D scores and probable clinical PPD as mean CES-D ≥ 16^18^.

Using the CES-D, the women also rated prospectively biweekly their antenatal depressive symptoms up to 14 times starting from 12 to 14 gestational week until delivery for women who gave birth to a preterm baby and until 38–39 gestational weeks for women who gave birth to a full-term baby. Of these biweekly CES-D measurements we identified the four consecutive measurements before childbirth: we have previously shown in this sample that in women who gave birth to a preterm baby, depressive symptoms begin increasing approximately 8 weeks before childbirth^30^, a timeframe which is captured by the four biweekly consecutive CES-D measurement points before childbirth.

#### Mental and behavioral disorders in children

We identified diagnoses of mental and behavioral disorders from the HILMO from the birth of the child between 11/07/2006 and 07/24/2010 to 12/31/2018. The HILMO includes primary and subsidiary diagnoses of all inpatient hospital treatments and outpatient visits in specialized medical care coded using the International Statistical Classification of Diseases and Related Health Problems-10 (ICD-10). HILMO is a valid tool for research^31^. We studied any mental and behavioral disorder (ICD-10: F00-F99) as the outcome.

### Covariates and effect modifiers

The following variables were included as covariates: maternal age at childbirth (years), smoking at any time during pregnancy (no/any smoking during pregnancy), mode of delivery (vaginal delivery/ elective Caesarean section/urgent or emergency caesarean section), child's sex (male/female) and age (years) at first mental and behavioral disorder diagnosis with data extracted from hospital records, MBR and/or HILMO; maternal education level (secondary or less (International Standard Classification of Education (ISCED) levels 0 to 5)/tertiary (ISCED levels 6 to 8) and alcohol use (no/any alcohol use during pregnancy) were self‐reported in early pregnancy. Smoking and alcohol use were combined into one variable representing smoking and/or alcohol use at any time during pregnancy (yes/no). Maternal mood disorder diagnoses before the childbirth were identified from HILMO (ICD-10 codes F32–F33, F341 since 1996 and ICD-9 codes 2961, 2968A, and 3004A between 1987–1995). Effect modifiers of preterm birth on maternal and child outcomes included mode of delivery (defined as above), maternal preeclampsia (ICD-10 codes O11, O14, O15), which was extracted from the hospital records, MBR and /or HILMO and verified by an expert jury comprising two medical doctors and a research nurse with expertise in obstetrics and gynecology, placenta previa (ICD-10 code O44), chorioamnionitis (ICD-10 code O41.1) and premature rupture of membranes (ICD-10 code O42), which were extracted from medical records, MBR and/or HILMO.

### Statistical analyses

Associations between preterm birth and maternal PPD symptoms levels were tested using linear regression and with probable clinical PPD using logistic regression.

To identify trajectories of maternal depressive symptoms between antenatal and postpartum stage, we conducted latent class analysis (LCA) based on their four antenatal and two postpartum CES-D scores. We compared solutions with two to six latent classes. Based on criteria for the optimal number of classes described by Kongsted and Nielsen^32^, the optimal solution was based on (1) goodness-of-fit criteria (Akaike Information Criterion [AIC], Bayesian Information Criterion [BIC]), (2) reasonable distribution of participants across subgroups (at least 10% of the sample), (3) high certainty of classification identified by posterior probabilities, and (4) clear clinical characteristics of the participants within each of the subgroups. Using the optimal LCA solution, we then tested whether the women who gave birth to a preterm and term baby differed in their odds of belonging to groups differing in their depressive symptoms trajectories identified by the LCA.

To study whether maternal PPD symptoms mediated the association between preterm birth and mental and behavioral disorders in the children, we used SPSS PROCESS macro with 5000 bootstrapped samples^33^. Before testing mediation, we ensured that the criteria for mediation were met, namely that the predictor, mediator and outcome variables were associated. Therefore, before proceeding to mediation analyses we also examined if preterm birth and maternal PPD symptoms were associated with mental and behavioral disorders in children by using Cox proportional hazards models. The proportionality assumptions were met (tests for the proportional hazards assumption yielded *p* values ≥ 0.07).

To study if mode of delivery, maternal preeclampsia, placenta previa, chorioamnionitis and premature rupture of membranes modified the effects of preterm birth on maternal PPD, depressive symptoms trajectories, and child mental and behavioral disorders, we included interaction terms between these variables and preterm birth into statistical models, followed by main effects of these variables.

We then examined if preterm birth and maternal PPD symptoms had additive effects on the hazard of mental and behavioral disorders in children by applying Cox proportional hazards models. For this analysis, we compared the hazards of mental and behavioral disorders in the children who were born at term and whose mothers reported no probable clinical PPD (referent) with (a) children born at term and whose mothers reported probable clinical PPD, (b) preterm children whose mothers reported no probable clinical PPD and (c) preterm children whose mothers reported probable clinical PPD.

We report the associations as unadjusted and adjusted for all covariates. Additionally, in examining the associations between preterm birth and PPD, we conducted sensitivity analyses in which we excluded women with a previous history of mood disorders diagnosed before childbirth. Statistical analyses were conducted by using SAS 9.4 (SAS Institute, Inc., Cary, NC, USA), Stata 15 (StataCorp. 2017. Stata Statistical Software: Release 15. College Station, TX: StataCorp LLC) and SPSS-IBM (Software, v.24.0 SPSS). All methods were carried out in accordance with relevant guidelines and regulations.

### Ethical standards

The authors assert that all procedures contributing to this work comply with the ethical standards of the relevant national and institutional committees on human experimentation and with the Helsinki Declaration of 1975, as revised in 2008.

## Supplementary Information

Supplementary Information 1.

Supplementary Information 2.

Supplementary Information 3.

Supplementary Information 4.

Supplementary Information 5.

Supplementary Information 6.

Supplementary Information 7.
