# Supplementary material for: Maternal postpartum depressive symptoms partially mediate the association between preterm birth and mental and behavioral disorders in children
Source: Sci Rep. 2022 Jan 18;12:947. doi: 10.1038/s41598-022-04990-w (PMC8766431; doi:10.1038/s41598-022-04990-w)
Supplement: Supplementary file 1 — Supplementary Information 1. [file 41598_2022_4990_MOESM1_ESM.pptx]

## Slide 1
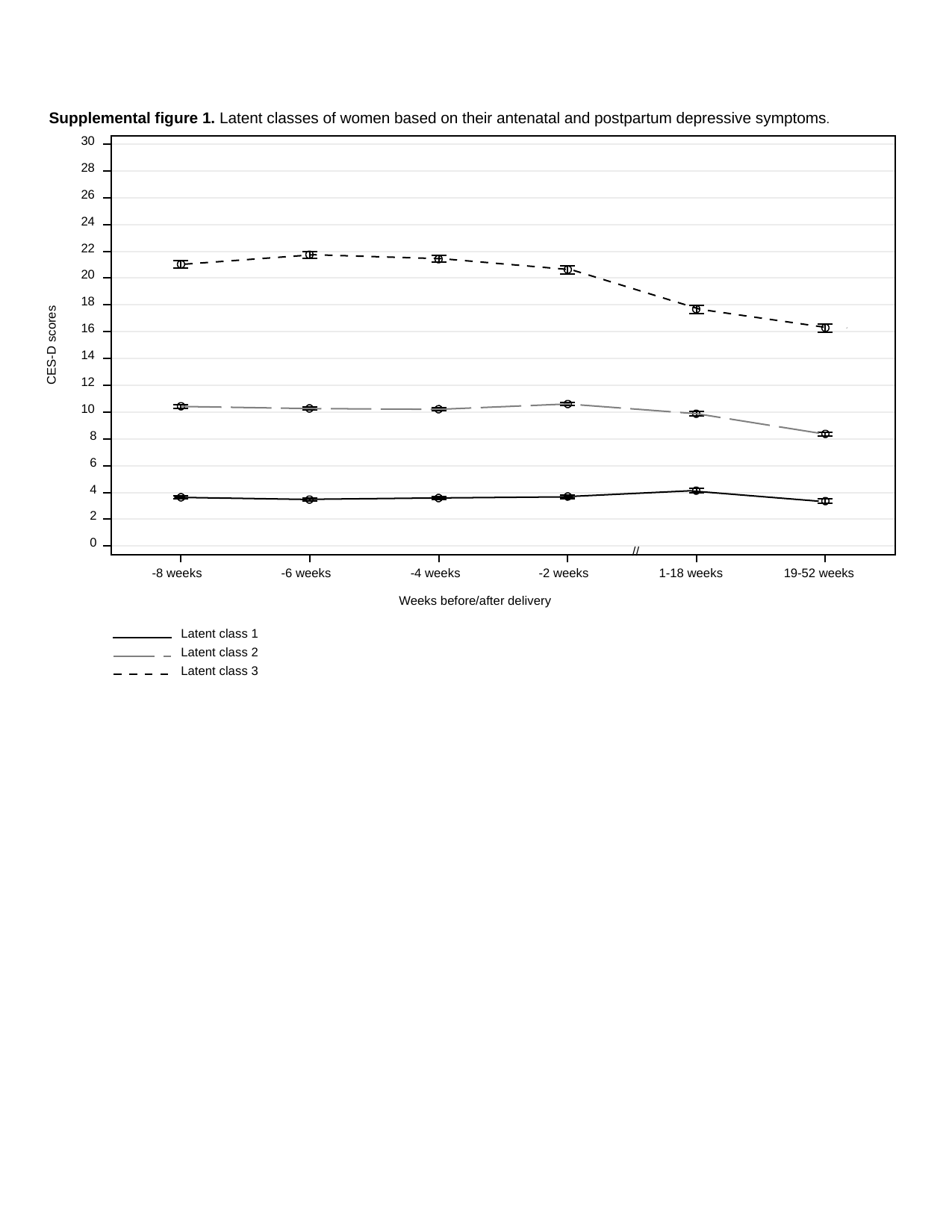

Supplemental figure 1. Latent classes of women based on their antenatal and postpartum depressive symptoms.
30
28
26
24
22
20
18
16
CES-D scores
14
12
10
8
6
4
2
0
//
-8 weeks	-6 weeks	-4 weeks	-2 weeks	1-18 weeks	19-52 weeks
Weeks before/after delivery
Latent class 1
Latent class 2
Latent class 3
