# Supplementary material for: Maternal postpartum depressive symptoms partially mediate the association between preterm birth and mental and behavioral disorders in children
Source: Sci Rep. 2022 Jan 18;12:947. doi: 10.1038/s41598-022-04990-w (PMC8766431; doi:10.1038/s41598-022-04990-w)
Supplement: Supplementary file 2 — Supplementary Information 2. [file 41598_2022_4990_MOESM2_ESM.docx]

| **ST1. Main characteristics of the cohort and cross-sectional studies that have examined associations between preterm birth and maternal postpartum depression (PPD).** | | | | | | | | | | | | | |
| --- | --- | --- | --- | --- | --- | --- | --- | --- | --- | --- | --- | --- | --- |
| **Citation** | **Country** | **Design** | **Number of preterm and term in sample** | **Source for gestation length and definition of preterm and term** | **PPD measure** | **Timing of PPD after delivery** | **Eligibility / Covariates** | **Result** | **Selection** | **Comparability** | **Outcome** | **Quality of evidence** | **Reported difference** |
| **Cohort studies** | | | | | | | | |  |  |  |  |  |
| Gungor et al. 2011 | Turkey | Cohort | 149 preterm  150 term | Source of gestational length is not specified  preterm (<37 GW)  term (≥37 GW) | BDI | 1-3 d | Eligibility: 17-44 years of age, no chronic illnesses, no fetal anomalities  Covariates: not included | Significant difference in PPD [preterm: M (SD) 9.35 (5.76), term M (SD) 7.28 (5.55), t=3.16, p (unadjusted)=0.002] | 2 | 0 | 2 | 4  Low | No |
| Brandon et al. 2011 | USA | Cohort | 29 preterm  31 full term | Medical records  preterm (35-36 GW)  full term (38-42 GW) | EPDS | birth; 1 mo | Eligibility: infants born 37 GW excluded, no multiples, preterm and term groups matched for ethnicity and mode of delivery  Covariates: not included | Significant difference in PPD [pooled estimate for birth and 3 mo (unadjusted) 2.24, S.E. 0.73] | 2 | 0 | 3 | 5  Low | Yes |
| Zanardo et al. 2011 | Italy | Cohort | 42 late preterm  40 term | Medical records  late preterm (34-36 GW)  term (not defined) | EPDS | 3-4 d | Eligibility: singleton, late preterm and term groups were matched for parity and delivery route  Covariates: not included | Significant difference in PPD [late preterm: M 9.5, SD 4.5, term: M 6.3 SD 3.9, p(unadjusted)=0.0008] | 2 | 0 | 2 | 4  Low | Yes |
| Gray et al. 2012 | Australia | Cohort | 86 preterm  96 term | Medical records  preterm (24-30 GW)  term (≥37 GW) | EPDS>12 | 4 mo | Eligibility: no multiple births greater than twins, births of which the other twin died, no major congenital anomalies in infant, infants not expected to live beyond hospital discharge and mothers who were not English speaking; preterm: treated at NICU, term: healthy, not treated in special care  Covariates: not included | No difference in PPD [preterm: 10 (11.6%), term:15 (15.6%), p (unadjusted) 0.44] | 2 | 0 | 3 | 5  Low | No |
| McDonald et al. 2013 | Canada | Cohort | 77 preterm  1,150 term | Mother-reported  preterm (34-36 GW)  term (≥38 GW) | EPDS≥13 | 4 mo | Eligibility: singleton,  37 GW excluded  Covariates from self-reports: history of depression, prenatal depression, prenatal anxiety, household income | No significant differences in PPD [OR (adjusted) 0.73 (0.24, 2.23)] | 1 | 2 | 3 | 6  Moderate | No |
| Braarud et al. 2013 | Norway | Cohort | 63 preterm  238 full-term | Medical records  preterm (30-36 GW)  full-term (37-42 GW) | EPDS | infant age: 3 mo; 6 mo; 9 mo | Eligibility: 30-42 GW, no acute or chronic neurological problems, known medical diagnoses, visual or auditory impairment or SGA (<-3SD) in infant  Covariates: not included | No significant difference in PPD [3 mo: t (unadjusted) −2.25, df 78.82, 95% CI −2.60 –0.16, p-value=; 6 mo: t (unadjusted) t −.84, df 282 95% CI −1.46–.59; 9 mo: t −.95, df 271, 95% CI −1.53–.54] | 2 | 0 | 3 | 5  Low | No |
| Pooler et al. 2013 | USA | Cohort | 9,764 preterm  34,563 term | Birth certificate  preterm (<37 GW)  term (≥37 GW) | PHQ-2 | 2-6 mo | Eligibility: alive infants residing with the mother  Covariates: demographic characteristics (maternal race and ethnicity, age at delivery, education and parity), risky behaviors (pregnancy intendedness, IPV during pregnancy, job loss and homelessness in the 12 months prior to delivery, binge drinking and smoking during pregnancy), WIC participation | Significant differences in PPD  AOR 1.29 (1.015, 1.44) | 3 | 1 | 2 | 6  Moderate | Yes |
| Herguner et al. 2013 | Turkey | Cohort | 35 VLBW  35 LBW  35 normal weight | Medical records  VLBW (<1500g)  normal weight (>2500 g) | EPDS>12 | 5 mo | Eligibility: not receiving medication for depression, no multiple birth, no congenital malformations in infant, mother aged 18-35 years, ability to read sufficiently and comprehend Turkish  Covariates: not included | Significant difference in PPD [VLBW: 15/35 (42.9%) vs. normal weight: 2/35 (5.7%), p=.000] | 2 | 1 | 3 | 6  Moderate | Yes |
| Helle et al. 2015 | Germany | Cohort | 111 VLBW  119 term | medical records  VLBW (<1500g)  term (≥34 GW) | EPDS ≥13,  BDI≥18,  SCID-I (mood disorders) | 4-6 wk | Eligibility: singleton and multiple  Covariates: history of psychiatric disorder not included in mother and father alone analyses, only included in analyses of both parents analyzed together | Significant differences in PPD of mothers in unadjusted analyses [(EPDS: OR 5.1 (2.2, 11.8), BDI: OR 18.4 (2.4, 141.5), SCID-I: OR 4.3 (1.2, 15.7)]; Adjusted estimates only reported in the analysis of both parents. | 1 | 0 | 3 | 4  Low | Yes |
| Bouras et al. 2015 | Greece | Cohort | 77 preterm  125 term | Medical personnel  preterm (<37 GW)  term (≥37 GW) | BDI | 1 mo | Eligibility: no previous diagnosis of mental disorders or drug use, preterm infant at least 1 week in NICU, term infant healthy, not requiring admission to NICU  Covariates: not included | Significant difference in PPD [(preterm: M(SD) 10.26 (7.88), term: M(SD) 7.06 (7.98) p=.001] | 1 | 1 | 2 | 4  Low | Yes |
| Cheng et al. 2016 | USA | Cohort | 450 very preterm  900 moderate / late preterm  4000 term | Birth certificate  very preterm (<32 GW)  moderate / late preterm (32-36 GW)  term (≥37 GW) | CES-D (12-item) ≥9 | 9 mo | Eligibility: no births to mother aged under 15 years, no adopted children, data required from both biological mothers and biological resident or non-resident fathers  Covariates: not included | No significant difference in PPD (above cutoff) [very preterm:14.9%, moderate/late preterm: 13.9, term: 14.1%, p (unadjusted) 0.93] | 3 | 0 | 2 | 5  Low | No |
| Pace et al. 2016 | Australia | Cohort | 149 very preterm  151 full term | Medical records  very preterm (<30 GW)  term (≥37 GW and < 2499g) | CES-D≥16 | shortly after birth; 6 mo | Eligibility: no congenital anomalies, infants unlikely to survive, do not speak English; term: not admitted to neonatal nursery  Covariates from self-reports: social risk (family structure, education of primary caregiver, occupation of primary income earner, employment status of primary income earner, language spoken at home, and maternal age at birth) and number or older siblings | No significant difference in PPD in models adjusted for covariates [OR (adjusted) not reported] | 2 | 1 | 3 | 6  Moderate | No |
| Warzecha et al. 2017 | Poland | Cohort | 57 preterm  299 term (5d);  16 preterm  90 term (6wk) | Mother-reported  preterm (<37 GW)  term (≥37 GW) | EPDS≥10 | <5 d;  6 wk | Eligibility: all births, fixed time-period, adequate knowledge of Polish  Covariates from self-reports: psychiatric history, demographic, satisfaction with perinatal care, relationship satisfaction with own mother, child’s father | No significant difference in PPD [OR (adjusted) not reported] | 2 | 0 | 2 | 4  Low | No |
| Youn et al. 2017 | Korea | Cohort | 37,296 preterm  1,231,834 term | Medical health insurance records preterm not defined  term not defined | ICD-10 F32, F33 | ≤12 mo | Eligibility: all births  Covariates: age, parity, multiple pregnancy, mode of delivery, induced labor, pre-eclampsia, GDM, placenta previa, placental abruption, uterine artery embolization, peripartum hysterectomy, previous depression | Significant difference in PPD [OR (adjusted) 1.41 (1.31, 1.52)] | 3 | 2 | 3 | 8  High | Yes |
| Silverman et al. 2017 | Sweden | Cohort | 5,388 early preterm  33,094 moderate/  late preterm  610,577 term | Medical birth register  early preterm (<32 GW)  moderate/late preterm (32-36 GW)  term (37-41 GW) | ICD-9, ICD-10* | ≤12 mo | Eligibility: all births  Covariates from medical birth register history of depression, year of delivery, maternal age at delivery, cohabitation with the father of the infant, hypertensive diseases, diabetic diseases, prolonged labor, mode of delivery, gestational age, birth weight for gestational age, congenital malformation, sphincter rupture | Significant differences in PPD [early preterm vs. term: RR(adjusted) 1.36(1.05, 1.75); moderate / late preterm vs. term: RR (adjusted) 1.20 (1.06, 1.36)] | 3 | 2 | 3 | 8  High | Yes |
| Harris et al. 2018 | USA | Cohort | 37 very preterm  47 full term | Medical records  very preterm (≤32 GW)  full-term (≥37 GW) | EPDS>10 | 85 d for very preterm, 2 d for term | Eligibility: no congenital anomalies; term: no medical complications, mother under 18 years, no drug use, do not speak English  Covariates: race, mode of delivery, siblings | Significant difference in PPD [very preterm: 11 (30%), term: 6 (13%), p (adjusted) 0.02] | 2 | 1 | 1 | 4  Low | Yes |
| Mehler et al. 2018 | Germany | Cohort | 60 preterm  30 full-term | Medical records  preterm (32-37 GW)  full-term (GW not reported) | EPDS 9-12  EPDS>12 | 2-10 d;  3 mo | Eligibility: no parental history of depression or other mental disorder, no malformations, small- or large-for-gestational-age infants, no full-term infants needing medical treatment, need to understand German language  Covariates: not included | No significant differences in PPD [EPDS 9-12, 2-10 days: preterm 12/60 (20%) vs. full-term 2/32 (6%), p=0.07; EPDS 9-12, 3 months: preterm 3/54 (6%) vs full-term 1/28 (4%), p=0.58; EPDS≥12, 2-10 days: preterm 4/60 (7%) vs. full-term 2/32 (6%), p=0.65; EPDS≥12, 3 months: preterm 3/54 (6%) vs full-term 4/28 (14%), p=0.18] | 1 | 1 | 3 | 5  Moderate | No |
| Koutra et al. 2018 | Greece | Cohort | 111 preterm  926 term | Medical records  preterm (<37 GW)  term (≥37 GW) | EPDS≥13 | 8 wk | Eligbility: no severe psychiatric disorder (i.e., schizophrenia, biopolar disorder), need to understand Greek, age >16 y, singleton  Covariates from self-reports: demographics, sensitivity analyses excluding women with EPDS≥13 at 28-32 GW | No significant differences in PPD [OR (adjusted) 1.25 (0.7, 2.2)] | 3 | 2 | 3 | 8  High | No |
| Meltzer-Brody et al. 2017 | Denmark | Cohort | 392,458 women  (134 preterm among 983 women with PPD) total number of preterm and term births not presented | Medical birth register preterm (ICD-10 O60, <37 GW)  term (≥37 GW) | ICD-10 F32, F33 (excluding F32.3) | ≤12 mo | Eligibility: no previous history of psychiatric illness, primiparous, singleton  Covariates from medical records: demographic, obstetric and pregnancy complications, psychiatric history of proband’s parents and child’s father | No significant differences in PPD [IRR (adjusted) 1.13 (0.94, 1.36)] | 3 | 2 | 3 | 8  High | No |
| Barber et al. 2021 | USA | Cohort | 73,475 term  9,685 MLPT  4,979 VPT  1,227 EPT | Birth certificate  preterm (<37 GW)  moderate or late preterm (32-37 GW)  very preterm (28-32 GW)  extremely preterm (<28 GW) | PHQ-2 | 2-4mo | Eligibility: age 18-45 years, no depression diagnosis before pregnancy, child is alive at the time of survey.  Covariates: race/ethnicity, maternal age, maternal education level, household income, maternal health insurance, marital status, gestational diabetes, gestational hypertension, parity, receipt of postpartum check-up, infant placed in neonatal intensive care unit after birth, infant currently alive, child currently living with participant, and presence of maternal stressful life events. | Significant difference in hopelessness [preterm vs. term: OR (adjusted) 1.24 (1.08, 1.43); MPLT vs term: OR (adjusted) 1.19 (1.0, 1.42);  VPT vs term: OR (adjusted) 1.28 (1.04, 1.58);  EPT vs. term: OR (adjusted) 1.81 (1.31, 2.49)] | 3 | 2 | 2 | 7  High | Yes |
| **Cross-sectional studies** | | | | | | | | |  |  |  |  |  |
|  | | | | | | | | |  |  |  |  |  |
| Bener, 2013 | Qatar | Cross-sectional | 170 preterm/  LBW  1,489 full-term | Medical personnel  preterm (≤37 GW)  LBW (<2500g)  full-term (does not define full-term) | DASS-21≥10 | ≤6 mo | Eligibility: preterm, LBW, full term  Covariates from interviews: parity, family support, baby gender, complicated pregnancy, complicated delivery, and gestational age | Significant difference in PPD (OR (adjusted) 2.0 (1.4, 2.9) | 3 | 1 | 1 | 5  Low | Yes |
| Leonard et al. 2014 | USA | Cross-sectional | 961 term  452 preterm | Mother-reported  preterm (<37 weeks or more than 3 weeks before the due date)  term (≥37 GW) | PHQ-2 | 2-4mo | Eligibility: has given singleton birth within the last 12 months, not pregnant at the time of filling in the PHQ-2  Covariates: not included | No difference in PPD  30.2% in preterms vs 26.3% in terms, p=0.13 | 2 | 1 | 1 | 4  Low | No |
| Enatescu et al. 2016 | Romania | Cross-sectional | 15 preterm  148 term | Medical records  preterm (< 37 GW)  term (GW not reported) | EPDS>12 | 6-8 wk | Eligibility: no history of prior psychiatric disorder (including the antenatal period)  **Covariates: not included** | Significant difference in PPD [(OR 7.2 (1.6, 32.1)] | 2 | 1 | 2 | 5 Low | Yes |
| Henderson et al. 2016 | UK | Cross-sectional | 42 very preterm  243 moderate / late preterm  4,176 term | Mother-reported  very preterm (<32 GW)  moderate / late preterm (32-36 GW)  term (≥37 GW including postterm) | EPDS>10 in Table EPDS>11 | 10 d; 1 mo; 3 mo | Eligibility: age younger than 16 years, infant death  Covariates: not included | No significant difference in PPD [10 d: very preterm 9 (22.0%), moderate / late preterm 94 (39%), term 1437 (34.7%); 1 mo: very preterm 4 (9.8%), moderate / late preterm 31 (12.9%), term 636 (15.4%); 3 mo: very preterm 1 (2.4%), moderate / late preterm 11 (4.6%), term 275 (6.6%)] | 4 | 0 | 0 | 4  Low | No |
| Liu et al. 2017 | China | Cross-sectional | 43 preterm  839 term | Mother-reported  preterm (<37 GW)  term (≥37 GW) | EPDS>10  usually cutoff is 10 or more | 4 wk | Eligibility: no history of mental illness or brain diseases  Covariates from self-reports: demographic, obstetric complications, delivery mode, infant birth weight and weight at 4 weeks, breastfeeding | No significant difference in PPD in adjusted models (effect size estimate=0.00, test statistics are not reported) | 2 | 2 | 0 | 4  Low | No |
| Cruise et al. 2018 | Ireland | Cross-sectional | 715 preterm  10,157 term | Mother-reported  preterm (25-36 GW)  term (≥37 GW) | CES-D (8 item)≥7 | 9 mo | Eligibility: all births  Covariates from self-reports: previous mental health problems, demographic, infant health and development, partner’s CES-D (8-item) score | Significant difference in PPD [(OR (adjusted) 1.35 (1.07, 1,70)] | 2 | 3 | 1 | 6  Low | Yes |
| *ICD-9: 296.20, 296.21, 296.22, 296.23, 296.3, 296.31, 296.32, 296.33, 296.34, 296.99, 301.1, 309.0, 311, 311.0, 648.40, 648.42, 648.44; ICD-10: F32, F320, F321, F322, F32.4, F32.8, F32.9, F33, F33.0, F33.1, F33.2, F33.3, F33.4, F33.8, F33.9, F34.0, F34.1, F34.8, F34.9, F38.0, F38.1, F38.8, F39, F53, F530, F53.1, F53.8, F53.9 (F32.3 only included as a covariate) | | | | | | | | |  |  |  |  |  |

For the cohort studies high quality was defined as a grade 7 or 8, Moderate quality was defined as a grade 5 or 6 and at least one point in the Selection, Comparability and Outcome sections, Low quality as a grade 4 or no points in either Selection, Comparability and Outcome sections.

For the cross-sectional studies high quality was defined as a grade 8, 9 or 10 with at least three points in the Selection, two points in the Outcome sections and at least one point in the Comparability section, Moderate quality was defined as a grade 6 or 7 with at least three points in the Selection, two points in the Outcome sections and at least one point in the Comparability section, Low quality was defined as a grade 5 or lower or less than three points in the Selection, two points in the Outcome sections or no points in the Comparability section.
