# Supplementary material for: Maternal postpartum depressive symptoms partially mediate the association between preterm birth and mental and behavioral disorders in children
Source: Sci Rep. 2022 Jan 18;12:947. doi: 10.1038/s41598-022-04990-w (PMC8766431; doi:10.1038/s41598-022-04990-w)
Supplement: Supplementary file 4 — Supplementary Information 4. [file 41598_2022_4990_MOESM4_ESM.docx]

| **ST3. Characteristics of the women who did provide the data on postpartum depressive symptoms (PPD) and those who did not** | | | |
| --- | --- | --- | --- |
|  | **Mean (SD) or N (%)** | |  |
|  | **Women who provided data on PPD**  **(N=3158)** | **Women who did not provide data on PPD**  **(N=1401)** | **P (comparison between groups)** |
| Maternal age at delivery, years | 31.8 (4.7) | 30.8 (5.2) | <0.0001 |
| Data not available | 0 | 0 |  |
| Education level |  |  | 0.08 |
| Upper secondary or less | 1278 (40.6%) | 457 (43.6%) |  |
| Tertiary | 1874 (59.4%) | 591 (56.4%) |  |
| Data not available | 6 (0.2%) | 353 (25.2%) |  |
| Smoking or alcohol use at any point during pregnancy |  |  | <0.0001 |
| No | 2446 (78.5%) | 218 (47.9%) |  |
| Yes | 672 (21.6%) | 237 (52.1%) |  |
| Data not available | 40 (1.3%) | 946 (67.5%) |  |
| Mode of delivery |  |  | 0.88 |
| Vaginal | 2608 (82.9%) | 1158 (82.7%) |  |
| Caesarean section | 538 (17.1%) | 242 (17.3%) |  |
| Data not available | 12 (0.4%) | 1 (0.1%) |  |
| Mood disorder diagnoses before childbirth |  |  | 0.0001 |
| No | 3015 (95.5%) | 1299 (92.7%) |  |
| Yes | 143 (4.5%) | 102 (7.3%) |  |
| Data not available | 0 | 0 |  |
| Preterm birth |  |  | 0.34 |
| No | 3033 (96.0%) | 1337 (95.4%) |  |
| Yes | 125 (4.0%) | 64 (4.6%) |  |
| Data not available | 0 | 0 |  |
|  | | | |
