# Supplementary material for: Maternal postpartum depressive symptoms partially mediate the association between preterm birth and mental and behavioral disorders in children
Source: Sci Rep. 2022 Jan 18;12:947. doi: 10.1038/s41598-022-04990-w (PMC8766431; doi:10.1038/s41598-022-04990-w)
Supplement: Supplementary file 5 — Supplementary Information 5. [file 41598_2022_4990_MOESM5_ESM.docx]

| ST4. Associations between covariates and maternal postpartum depressive symptoms (PPD) and mental and behavioral disorders in children. | | | | | | |
| --- | --- | --- | --- | --- | --- | --- |
| Covariates | PPD symptoms | | | Mental and behavioral disorders in children | | |
|  | Mean difference (SD units) | 95% Confidence Interval | P | Hazard Ratio | 95% Confidence Interval | P |
| Maternal age at delivery (years) | -0.01 | -0.02, -0.01 | 0.004 | 1.00 | 0.98, 1.02 | 0.82 |
| Maternal education (tertiary vs. secondary and lower) | -0.10 | -0.18, -0.03 | 0.004 | 0.54 | 0.44, 0.67 | <0.0001 |
| Maternal smoking and/or alcohol use at any time during  pregnancy (yes vs. no) | 0.03 | -0.05, 0.12 | 0.48 | 1.16 | 0.90, 1.49 | 0.26 |
| Delivery mode (caesarian section vs. vaginal) | 0.15 | 0.06, 0.24 | 0.001 | 0.99 | 0.75, 1.33 | 0.99 |
| Maternal mood disorder diagnoses before  childbirth (yes vs. no) | 0.70 | 0.53, 0.86 | <0.0001 | 2.65 | 1.86, 3.76 | <0.0001 |
| Maternal antenatal depressive symptoms (SD) | 0.71 | 0.68, 0.74 | <0.0001 | 1.39 | 1.23, 1.56 | <0.0001 |
| Child sex (boy vs. girl) | 0.07 | 0.00, 0.14 | 0.06 | 2.24 | 1.77, 2.83 | <0.0001 |
