# Supplementary material for: Maternal postpartum depressive symptoms partially mediate the association between preterm birth and mental and behavioral disorders in children
Source: Sci Rep. 2022 Jan 18;12:947. doi: 10.1038/s41598-022-04990-w (PMC8766431; doi:10.1038/s41598-022-04990-w)
Supplement: Supplementary file 6 — Supplementary Information 6. [file 41598_2022_4990_MOESM6_ESM.docx]

| ST5. Criteria used in Latent Class Analyses for identifying the most optimal solution to group women based on their antenatal-to-postpartum depressive symptoms. | | | | | |
| --- | --- | --- | --- | --- | --- |
| **Criteria:** | Number of groups based on the Latent Class Analyses | | | | |
|  | 2 | 3 | 4 | 5 | 6 |
| **Goodness-of-fit** |  |  |  |  |  |
| Akaike Information Criterion | 40605.23 | 38185.23 | 37004.73 | 36545.32 | 36268.75 |
| Bayesian Information Criterion | 40720.71 | 38343.25 | 37205.29 | 36788.43 | 36554.41 |
| **Number (%) of participants in groups** |  |  |  |  |  |
| 1 | 1477 (46.8%) | 633 (20.0%) | 360 (11.4%) | 295 (9.3%) | 71 (2.3%) |
| 2 | 1681 (53.2%) | 1696 (53.7%) | 1157 (36.6%) | 856 (27.1%) | 346 (11.0%) |
| 3 |  | 829 (26.3%) | 1236 (39.1%) | 1136 (36.0%) | 933 (29.5%) |
| 4 |  |  | 405 (12.8%) | 696 (22.0%) | 1052 (33.3%) |
| 5 |  |  |  | 175 (5.5%) | 614 (19.4%) |
| 6 |  |  |  |  | 142 (4.5%) |
| **Certainty of classification by posterior probability** | 0.94  (SD 0.12) | 0.91  (SD 0.14) | 0.89  (SD 0.15) | 0.85  (SD 0.16) | 0.84  (SD 0.16) |
| **Clinical relevance** | Clearly defined groups with consistently low and consistently high antenatal-to-postpartum depressive symptoms | Clearly defined groups with consistently low, consistently moderate and consistently high antenatal-to-postpartum depressive symptoms | Clearly defined groups with consistently high and consistently moderate antenatal-to-postpartum depressive symptoms, two groups with consistently low antenatal-to-postpartum depressive symptoms are not clearly different | Two groups with consistently low antenatal-to-postpartum depressive symptoms are not clearly different, clearly defined group with consistently moderate antenatal-to-postpartum depressive symptoms, two groups with consistently high antenatal-to-postpartum depressive symptoms are not clearly different | Three groups with consistently low antenatal-to-postpartum depressive symptoms are not clearly different, clearly defined group with consistently moderate antenatal-to-postpartum depressive symptoms, two groups with consistently high antenatal-to-postpartum depressive symptoms are not clearly different |
|  | Clinically relevant. Easy to identify women at risk | Clinically relevant. Easy to identify women at risk, more targeted identification of risk as compared to the two group solution | Clinical relevance of segregating the groups that do not distinctly differ from each other is not clear. LCA with 4-6 groups can be scaled down to the 3 groups solution. | | |
