# Supplementary material for: Maternal postpartum depressive symptoms partially mediate the association between preterm birth and mental and behavioral disorders in children
Source: Sci Rep. 2022 Jan 18;12:947. doi: 10.1038/s41598-022-04990-w (PMC8766431; doi:10.1038/s41598-022-04990-w)
Supplement: Supplementary file 7 — Supplementary Information 7. [file 41598_2022_4990_MOESM7_ESM.docx]

| ST6. Interactions between preterm birth and obstetric complications and mode of delivery on maternal postpartum depressive symptoms (PPD), latent classes of women based on antenatal and PPD symptoms and mental and behavioral disorders in children in a follow-up from birth until 8.4-12.8 years of age | | | | |
| --- | --- | --- | --- | --- |
| Interaction terms*  preterm birth and | Level of PPD symptoms | Latent classes of women based on antenatal and PPD symptoms | | Mental and behavioral disorders in children |
|  |  | Consistently moderate versus low | Consistently high versus low |  |
|  | P | P | P | P |
| Preeclampsia |  |  |  |  |
| No | Ref. | Ref. | Ref. | Ref. |
| Yes | 0.07 | 0.87 | 0.45 | 0.59 |
| Premature rupture of membranes |  |  |  |  |
| No | Ref. | Ref. | Ref. | Ref. |
| Yes | 0.28 | 0.22 | 0.34 | 0.58 |
| Delivery mode |  |  |  |  |
| Vaginal delivery | Ref. | Ref. | Ref. | Ref. |
| Elective Caesarian section | 0.29 | 0.97 | 0.95 | 0.35 |
| Urgent/emergency Caesarian section | 0.51 | 0.96 | 0.94 | 0.77 |
| *All interactions are tested in the presence of main effects  Models are adjusted for maternal age at delivery, education, smoking and/or alcohol use at any time during pregnancy, and child sex; Models of preeclampsia and premature rupture of membranes are also adjusted for delivery mode. | | | | |
